# Transcript levels of members of the SLC2 and SLC5 families of glucose transport proteins in eel swimbladder tissue: the influence of silvering and the influence of a nematode infection

**DOI:** 10.1007/s10695-017-0456-y

**Published:** 2018-01-11

**Authors:** Gabriel Schneebauer, David Mauracher, Birgit Fiechtner, Bernd Pelster

**Affiliations:** 1 Institute of Zoology, Leopold-Franzens-Universität Innsbruck, Technikerstr.25, 6020 Innsbruck, Austria; 2 Center for Molecular Biosciences, University of Innsbruck, Innsbruck, Austria

**Keywords:** Swimbladder, European eel, Glucose transport, Metabolism, mRNA expression, Transcription, Gas gland, Rete mirabile, Silvering, Anguillicola crassus

## Abstract

The rate of glucose metabolism has been shown to be correlated to glucose uptake in swimbladder gas gland cells. Therefore, it is assumed that in the European eel silvering, i.e., the preparation of the eel for the spawning migration to the Sargasso Sea, coincides with an enhanced capacity for glucose uptake. To test this hypothesis expression of all known glucose transport proteins has been assessed at the transcript level in yellow and in silver eels, and we also included *Anguillicola crassus* infected swimbladders. Glucose uptake by rete mirabile endothelial cells could be crucial for the countercurrent exchange capacity of the rete. Therefore, this tissue was also included in our analysis. The results revealed expression of ten different members of the *slc2* family of glucose transporters, of four *slc5* family members, and of *kiaa1919* in gas gland tissue. Glucose transporters of the *slc2* family were expressed at very high level, and *slc2a1b* made up about 80% of all *slc2* family members, irrespective of the developmental state or the infection status of the eel. Overall, the *slc5* family contributed to only about 8% of all detected glucose transport transcripts in gas gland tissue, and the *slc2* family to more than 85%. In rete capillaries, the contribution of sodium-dependent glucose transporters was significantly higher, leaving only 66% for the *slc2* family of glucose transporters. Neither silvering nor the infection status had a significant effect on the expression of glucose transporters in swimbladder gas gland tissue, suggesting that glucose metabolism of eel gas gland cells may not be related to transcriptional changes of glucose transport proteins.

## Introduction

Gas secretion in the swimbladder is dependent on glucose metabolism, resulting in the production of lactic acid in the glycolytic pathway, and of CO_2_, generated in the pentose phosphate shunt (Pelster and Scheid 1993; Walsh and Milligan 1993; Pelster et al. 1994; Pelster 1995b). Along the partial pressure gradient, CO_2_ diffuses from gas gland cells into the swimbladder, contributing by about 20% to newly secreted gas (Kobayashi et al. 1990), and into the blood, causing an acidification of the blood during passage of the gas gland cells. Similarly, lactic acid is released into the blood, adding to the acidification (Pelster 1995a). The acidification of the blood during passage of the gas gland tissue switches on the Root effect, so that oxygen is released from the hemoglobin and diffuses into the swimbladder along the partial pressure gradient (Pelster and Weber 1991; Pelster and Randall 1998; Pelster 2001). Accordingly, oxygen and CO_2_ are the main gases secreted into the swimbladder in the European eel (Kobayashi et al. 1990).

Gas secretion into the swimbladder therefore is dependent on glucose uptake from the blood, because internal glycogen stores do not appear to be of any importance (Pelster 1995b). A comparison of transcript levels of glucose transport proteins GLUT1-4 in various tissues of Atlantic cod *Gadus morhua* revealed that the GLUT1 transport protein is expressed in most tissues examined, and *glut1* expression accounted for 97.6% of the glucose transporter transcripts in gas gland tissue (Hall et al. 2014). Furthermore, in gas gland tissue *glut1* expression was several fold higher than in other tissues (brain, heart, red blood cells), and the elevated level of *glut1* transcript expression in gas gland cells coincided with an elevated rate of glucose consumption, as determined from ^3^H_2_O production from labeled glucose (Hall et al. 2014). In gas gland tissue, a linear correlation between *glut1* transcript expression and glucose metabolism was observed, and lactate production of gas gland cells was 11-fold higher than in cardiac muscle cells, demonstrating the importance of glucose uptake and lactate production in gas gland tissue. A recent detailed study on glucose metabolism in gas gland tissue of the Atlantic cod confirmed the critical importance of the GLUT1 transport protein for the rate of glucose utilization (Clow et al. 2016).

Searching the fugu *Takifugu rubripes* database for glucose transporter genes, 22 genes for facilitative glucose transporters and seven genes for sodium-dependent glucose cotransporters were detected (Munakata et al. 2012). Using RT-PCR, 7 out of the 22 genes for facilitative glucose transporters were found to be expressed at higher levels, but only *glut1a* and *glut6* could be detected using in situ hybridization. Expression of sodium-dependent glucose transporter genes was very low and not pursued any further (Munakata et al. 2012).

The basics of swimbladder metabolism of the European eel have been obtained by analyzing yellow eels, i.e., the juvenile stage of the eel living in the European freshwater system. In a process named silvering, the eel prepares for the migration back to the spawning grounds in the Sargasso Sea. During this migration, eels perform daily vertical migrations and therefore are exposed to very high hydrostatic pressures up to 100 atm (Aarestrup et al. 2009; Righton et al. 2016; Schabetsberger et al. 2016). During silvering, the retia mirabilia*,* sophisticated and very effective countercurrent systems, are significantly enlarged, indicating an improvement of the countercurrent concentrating ability. In addition, the performance of the swimbladder is improved by increasing swimbladder wall thickness and guanine deposition (Kleckner 1980a; Kleckner 1980b; Yamada et al. 2001). In the American eel *Anguilla rostrata*, a five-fold increase in the rate of gas deposition has been recorded in silver eels (Kleckner 1980a). It therefore is assumed that these silvering induced changes are connected to a significant improvement in swimbladder function (Sebert et al. 2009; Righton et al. 2012). The rate of gas secretion has been shown to be correlated to the rate of glucose uptake (Pelster and Scheid 1993) and to the rate of lactate production (Pelster 1995b). Accordingly, an improvement of swimbladder function in silver eels will require an increased uptake of glucose.

Analysis of the transcriptional changes that occur during silvering in gas gland tissue surprisingly did not provide any indication for an enhanced glycolytic activity in silver eels, but for *glut5* and *6,* elevated transcript levels were detected in silver eel gas gland tissue as compared to yellow eel gas gland. For *glut1*, the most important glucose transporter in Atlantic cod gas gland, however, no difference was detected (Pelster et al. 2016). For European eel, the importance of the various glucose transport proteins has not been assessed, and it therefore appears possible that, in contrast to the Atlantic cod, in the eel swimbladder glucose metabolism is influenced or perhaps even controlled by several glucose transport proteins.

In addition to the gas gland cells, the retia mirabilia, representing a fantastic countercurrent system, are crucial for swimbladder function. Rete capillaries are not only permeable to gas molecules, metabolites like lactate are also concentrated in the rete and this requires transport proteins. Most transport processes at some point require energy, derived from glucose metabolism. Surprisingly, analysis of glucose metabolism of rete capillaries revealed that glucose is almost completely converted to lactate, although rete tissue also is exposed to high oxygen levels (Rasio 1973). In the present study, we therefore searched the genome of the European eel for possible members of the glucose transporter families SLC2 (GLUT-transporters), SLC5 (Na^+^-dependent glucose transport proteins, SGLT), and KIAA1919 (NaGLT1) and determined the transcript level of these proteins in gas gland tissue. Members of the SLC2 family are facilitative glucose transporters, allowing glucose transport along a concentration gradient (Mueckler and Thorens 2013), while members of the SLC5 family and also KIAA1919 couple glucose transport to the downhill sodium transport (Wright and Turk 2004). In addition to gas gland tissue, we also analyzed the capillary tissues of the rete mirabile for glucose transporter transcript levels to test the hypothesis that rete membranes and gas gland cells use a different set of glucose transport proteins, allowing for an independent control of glucose metabolism in these two tissues. Because following the introduction of the nematode *Anguillicola crassus* into the European freshwater system the swimbladder of a large fraction of European yellow and silver eels is infected with this nematode (Moravec 1992; Schabuss et al. 2005), we also included gas gland tissue of infected yellow and silver eels in our analysis. Due to the hematophagous and histophagous feeding habits of the nematode, it appears possible that the infection of the swimbladder has an influence on glucose uptake of the gas gland tissue. A comparison of the gas gland transcriptome of uninfected and infected silver eels with uninfected yellow eels suggested that the nematode may be able to modify transcriptional activity of gas gland cells (Pelster et al. 2016).

## Materials and methods

### Animals

All experiments were performed with female European eels (*Anguilla anguilla*). Yellow and silver eels were caught by local fishermen in Lake Constance, Bregenz, Austria, and kept in an outdoor freshwater basin at the Institute of Zoology at the University of Innsbruck, not exceeding 3 weeks. Two days prior to sampling, fish were transferred into an indoor freshwater aquarium. Yellow eels were also caught by local fishermen in the open water of the River Elbe, close to Winsen (Luhe), Germany, and kept in an outdoor basin with a freshwater supply at the Thünen Institute of Fisheries Ecology, Ahrensburg, Germany, until sampling, not exceeding 7 days. Table 1 shows the morphometrics of uninfected and infected yellow and silver eels chosen for the experiments. The silver index was calculated according to Durif et al. (2005), and the ocular index according to Pankhurst (Pankhurst 1982).

**Table 1:** Morphometrics, silvering index calculated according to Durif et al. (2005) and ocular index calculated according to Pankhurst (1982) of uninfected and infected yellow and silver eels. Overall mean values ± S.E.M.; *N* = 5

		Yellow uninfected	Silver uninfected	Yellow infected	Silver infected
Body mass	(g)	379,80 ± 110.54	906.00 ± 68,30	235.60 ± 34.40	870.00 ± 76.61
Body length	(cm)	64.20 ± 5.86	85.50 ± 2,52	52.80 ± 2.44	83.40 ± 2.50
Pectoral fin length	(mm)	26.68 ± 2.67	43.02 ± 0.89	22.92 ± 1.57	41.74 ± 2.33
Horizontal eye diameter	(mm)	6.88 ± 0.52	9.87 ± 0.32	5.94 ± 0.43	9.94 ± 0.28
Vertical eye diameter	(mm)	6.08 ± 0.32	9.32 ± 0.45	5.80 ± 0.37	8.96 ± 0.33
Silver index		2.60 ± 0.24	4.50 ± 0.34	2.40 ± 0.25	4.20 ± 0.49
Ocular index		5.16 ± 0.25	8.53 ± 0.45	5.24 ± 0.47	8.46 ± 0.48

### Tissue preparation

Eels were anesthetized with neutralized tricaine methanesulfonate (MS-222; 0.1 g l^−1^) and subsequently decerebrated and spinally pithed. The abdominal wall was opened ventrally, and the swimbladder was carefully exposed. The artery and vein supplying blood to and removing it from the rete mirabile were occlusively cannulated using PE50 catheters. Rete mirabile and swimbladder tissue were perfused with heparinized (No. H3393; Sigma-Aldrich Co. LLC., St. Louis, MO, USA; 100 i.U. ml^−1^) Ringer’s solution, which contained (in mmol l^−1^) NaCl, 124; KC1, 5; MgSO_4_ (7 H_2_O), 0.9; CaCl_2_ (2 H_2_O)_,_ 1.1; NaHCO_3_, 10; Glucose, 5; to clear the tissue from blood cells (Pelster et al. 1989). After removal of blood cells, the two retia mirabilia and the gas gland tissue were carefully dissected, quickly dried on absorbent paper, transferred into 1 ml RNAlater™ solution (Invitrogen by Thermo Fisher Scientific Inc., Waltham, MA, USA), and immediately shock frozen in liquid nitrogen. Following perfusion, tissue preparation and cleaning took no more than 2 min. Tissues were then stored at − 80 °C until further use.

After completing tissue preparation, the number of nematodes isolated from infected swimbladders was counted. Eels with no or one small (length about 4 mm) *Anguillicola crassus* inside the swimbladder without visible modifications of the swimbladder wall were considered as uninfected. In these eels, after peeling off the connective tissue the swimbladder wall was transparent and thin. Heavily infected eels (Pelster et al. 2016) had between 9 and 54 *Anguillicola crassus* inside the swimbladder. In these fish, the swimbladder contained exudate, was comparatively small and thick walled, and had an opaque appearance. For each group, uninfected yellow, infected yellow, uninfected silver, and infected silver, five animals were analyzed. Rete mirabile tissue was sampled from yellow uninfected (*N* = 3), silver uninfected (*N* = 5), and silver infected eels (*N* = 5). Tissue sampling was performed in compliance with the Austrian law and the guidelines of the Austrian Federal Minister for Education, Arts and Culture.

### Isolation of total RNA and preparation of cDNA

Swimbladder and rete mirabile tissue was homogenized in Precellys tubes CKMix (No. KT03961-1-009.2), with a Precellys 24 homogenizer (Bertin Technologies, France) (2 × 30 s at 6000 rpm, 45 s break). Total RNA was isolated using the Qiagen miRNeasy® Mini Kit (No. 217004; Qiagen GmbH, Hilden, Germany) according to the manufacturer’s instructions. Quality of extracted RNA was assessed by electrophoresis on a 1.5% agarose-gel, confirming the integrity of 18S and 28S RNA-bands. RNA concentration was determined using the Quant-iT™ RiboGreen® RNA Assay Kit (No. R11490; Invitrogen by Thermo Fisher Scientific Inc., Waltham, MA, USA) and a VICTOR™ X4 Multilabel Plate Reader (PerkinElmer, Inc., Waltham, MA, USA). A total of 450 ng RNA was applied for first strand cDNA synthesis. The RNA was preincubated with 2.5 μl Random Hexamer Primer (No. SO142, Thermo Fisher Scientific Inc., Waltham, MA, USA) at 70 °C for 5 min before the RT reaction was set up using RiboLock™ RNase Inhibitor, 10 mM dNTP Mix, and RevertAid H Minus Reverse Transcriptase in 50 μl approaches (No. EO0381, No. R0191, and No. EP0451, respectively, Thermo Fisher Scientific Inc., Waltham, MA, USA). For each tissue sample, a cDNA synthesis without reverse transcriptase (noRT) was performed to detect DNA contaminations in the final qPCR reaction.

### Primer design and preparation of standard curves

Possible members of the glucose transporter families SLC2 (GLUT-transporters) and SLC5 (SGLT-transporters) and the glucose transporter KIAA1919 (NaGLT1) (Horiba et al. 2003) were identified by searching the draft genome sequence of European eel, *Anguilla anguilla*, (Henkel et al. 2012). Predicted cDNAs are available at http://www.zfgenomics.com/sub/eel.

This search identified 12 possible members of the *slc2* family of glucose transporters, five possible members of the *slc5* family of glucose transporters, and the sodium-dependent glucose transporter *kiaa1919*. The sequences g8232 and g18545 each represent discrete gene species of glucose transporter *slc2a1* (later referred to as *slc2a1a* and *slc2a1b*), and the sequences g13360 and g15843 each represent discrete gene species of the glucose transporter *slc2a11* (later referred to as *slc2a11a* and *slc2a11b*). For all identified possible glucose transporters, primers were designed using Primer Express© Software for Real-Time PCR3.0.0 (Applied Biosystems, Foster City, CA, USA). Primers were purchased from Microsynth (Balgach, Switzerland). All primer pairs, the gene numbers of the corresponding predicted cDNAs, and the aliases are listed in Table 2. For three transporters (*slc2a1a*; *slc2a9*; *slc5a2*), the initially designed primer pairs did not result in reliable amplification of a defined fragment. Therefore a second set of primers was designed (Table 2). Again, no defined fragment could be amplified. We therefore assumed that these transporters are only expressed with a very low copy number so that a reliable quantification is not possible.

**Table 2:** Aliases, gene numbers and sequences of forward and revers primers of all glucose transporters analyzed in this study

Glucose transporter	Aliases	Corresponding cDNA	Primer forward	Primer reverse
*kiaa1919*	*naglt1*, *naglut*	g11656	TGCAGTCGCTGGCTAATCAC	CATCCCCAGTCCGAGAAAAG
*slc2a1a*	*glut1*, *gtr1*	g8232	GGCACTGAGGACGTGAGTGA	TCCATCGCCATCTTCAGACTCT
*slc2a1b*	*glut1*, *gtr1*	g18545	GCATCAACGCCGTCTTCTACT	CTGGGCCACTCCTGCTTTC
*slc2a2*	*glut2*, *gtr2*	g6508	TTCGGTGGTGCTGGTTGA	CCCCCATATCCAATCAGAGTCA
*slc2a3*	*glut3*, *gtr3*	g12848	CAAGGAGGAAGAGGCACAGAA	CGCTCACATCCTCAGAACCA
*slc2a4*	*glut4*, *gtr4*	g3699	CATGTGTTTGTGTGCCATCATC	ACGGCACGCTTTCCAAGAG
*slc2a5*	*glut5*, *gtr5*	g21637	CCCGGAAACCAAAAACAAGA	CCCATTTCTCTCCGCAAACA
*slc2a6*	*glut6*, *gtr6*	g25279	CATGCCCTGCCCTCAATG	GGGTCAAAGGGTGTGGTCTGT
*slc2a8*	*glut8*, *gtr8*	g7936	ACCCCCGGTTGCAGTTG	GTCACCACAGACCCAAACCAT
*slc2a9*	*glut9*, *gtr9*	g21280	GGGTTGCGTGATCGGAATC	AGGGAACTCCAGCTGGTCCTA
*slc2a10*	*glut10*, *gtr10*	g6928	CCCGCTGGTCTGAGAGGAA	ATTAGCTGCCCAGTTGAAGCA
*slc2a11a*	*glut11*, *gtr11*	g13360	CAGAGGAACTGCTCACCTTGCT	GCCCGCCAATGGTGAAG
*slc2a11b*	*glut11*, *gtr11*	g15843	CTGTGCTGGCAGTGTTTAGCA	CCGGGCCAGAATGATCATC
*slc5a1*	*sglt1*	g18418	AAGCTGCTGCCCATGTTCAT	TCTGTGTACAGGATGCGACTGAT
*slc5a2*	*sglt2*	g14486	TGGTTCTGCGGCCTGAGT	CGACCTCCTGCTCCGTAAGT
*slc5a9*	*sglt4*	g9021	CCACCAATCACCGCTGTCT	CTGCTCATTGACACGTTTCCA
*slc5a10*	*sglt5*	g2925	CACCCGTCACTGCCATCTT	GAATGCTCCCGCCTCGTT
*slc5a11*	*sglt6*, *smit2*, *kst1*	g8799	GGATACCGCGTGCTTGCT	GGTTGCCGTTGACGAAGTG
*slc2a1a*	*glut1*, *gtr1*	g8232	GGGAGGCGTAAATCCATGCT	CAAAGCTGAGAAGCCCATCAG
*slc2a9*	*glut9*, *gtr9*	g21280	GCTGGGTGGTCGTAATGTTGT	TGCATACGGCAGGAGACCTT
*slc5a2*	*sglt2*	g14486	TTCTCTGCATCGTGTCCATCA	GCTCTGGGCAGCCTGAAC

A primer matrix with four different concentration pairs of the forward and reverse primer (3 × 10^−6^ and 9 × 10^−6^ mol l^−1^ for each primer) was used to identify the optimal primer concentration for the amplification reaction. The resulting PCR products of the primer matrix were purified using the QIAquick® PCR Purification Kit (No. 28106; Qiagen GmbH, Hilden, Germany); DNA concentration was determined using the Quant-iT™ PicoGreen® dsDNA Assay Kit (Invitrogen by Thermo Fisher Scientific Inc., Waltham, MA, USA) and used to create 7-point dilution series from 10^−9^ to 10^−3^ mol l^−1^ to calculate a standard curve for each glucose transporter.

### qPCR assays

Quantitative real-time PCR was performed using the Power SYBR® Green PCR Master Mix (Applied Biosystems™ by Thermo Fisher Scientific, Inc., Waltham, MA, USA). Each reaction contained 1 μl ultrapure water, 1 μl BSA, 1 μl cDNA (representing 9 ng of input RNA), 1 μl forward and 1 μl reverse primer, and 5 μl SYBR® Green and was assessed on a QuantStudio 3 Real-Time PCR System (Thermo Fisher Scientific Inc., Waltham, MA, USA), with the following cycling conditions: 2 min at 50 °C, 10 min at 95 °C, 40 cycles of 15 s at 95 °C, and 1 min at 60 °C, and a final melting curve from 60 to 95 °C to identify possible primer dimers and verify reaction specificity. Each sample was measured in triplicates. Each tissue was analyzed on a single plate with additional noRT and no template controls to test for DNA contaminations. On all plates, glucose transporter *slc2a1b* was measured in 1 μl of a defined cDNA pool and used as a calibrator. *C*_T_ levels for all other glucose transporters were calculated relative to this calibrator, so that a quantitative comparison between different plates was possible. Following this normalization, the *C*_T_-values were used for determination of the absolute copy number of the glucose transporter transcripts based on the respective standard curves. Copy numbers were calculated per 10 ng total RNA. Occasionally, due to varying primer efficiencies, copy numbers had to be corrected using a correction factor calculated according to Pérez et al. (2013).

### Statistics

Data are presented as mean ± S.E.M. with *N* giving the number of animals analyzed. Outliers were detected using IBM SPSS Statistics Version 24.0 (IBM Corp., Armonk, NY, USA). Shapiro-Wilk tests were performed using SigmaPlot 13.0 (Systat Software, Inc., San Jose, CA, USA), to test data of experimental groups for normal distribution. ANOVA on Ranks with subsequent Holm-Sidak or Dunn’s post hoc multiple comparison tests were performed to test for statistically significant differences between the experimental groups. Significance of differences was accepted when *P* < 0.05.

## Results

Using the primer sets designed on the basis of the eel genome, we obtained the expected amplicons for all glucose transport family members except *slc2a1a*, *slc2a9*, and *slc5a2*.

**Fig. 1:**
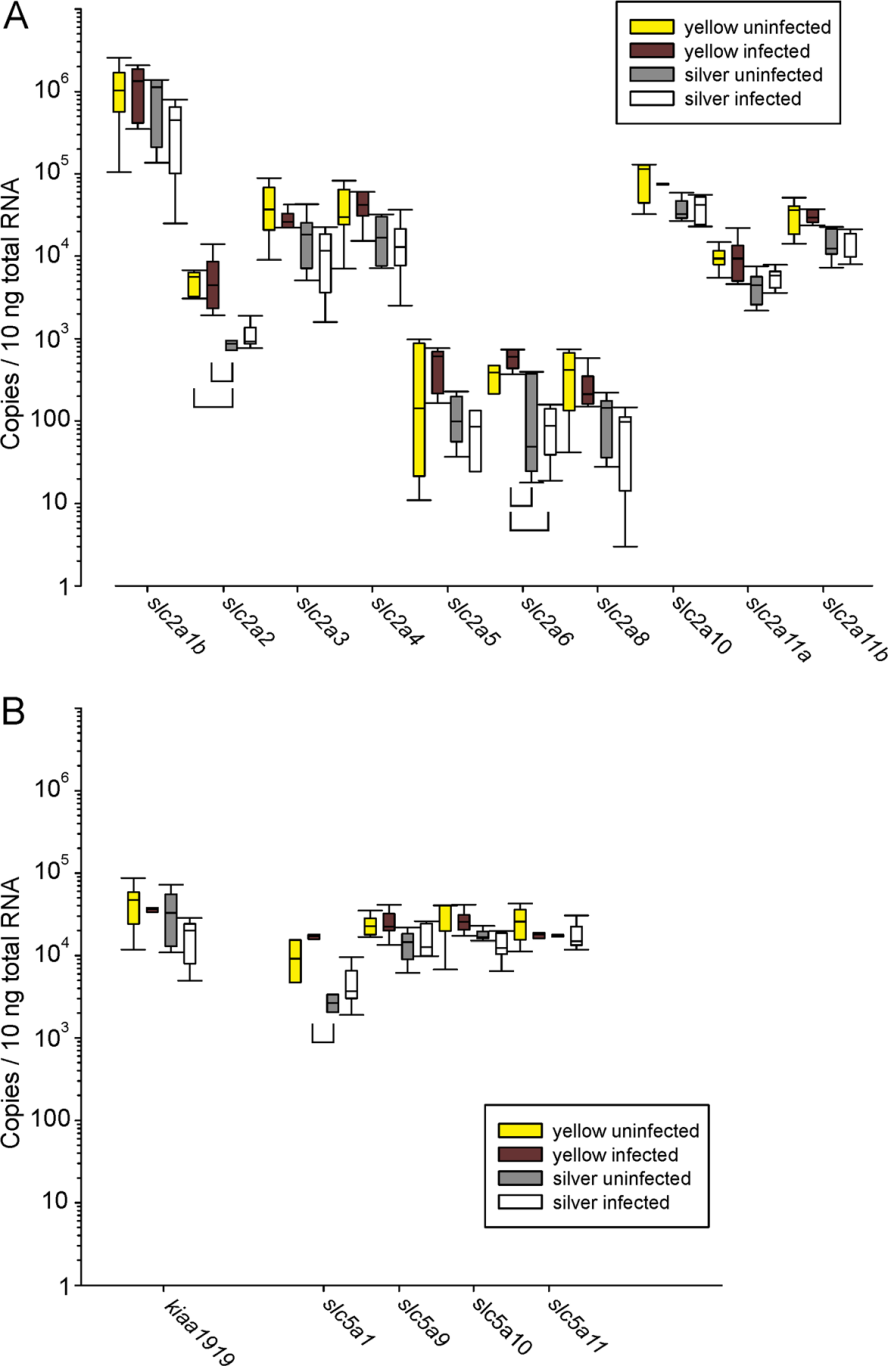
Number of mRNA copies of various members of the *slc2* (**a**) and the *slc5* (**b**) family of glucose transporters, and of *kiaa1919* (**b**) per 10 ng total RNA in gas gland tissue of uninfected and infected yellow and silver eels. Due to the large differences in the copy number, data are presented on a log scale. *N* = 5 for each group. Bracket indicates significant differences between groups, *P* < 0.05

In uninfected yellow eel gas gland tissue, the member of the *slc2* family of glucose transporters with the highest transcript level was *slc2a1b*, expressed with 1.16 ± 0.48 × 10^6^ copies per 10 ng of total RNA (Fig. 1a). In uninfected silver eel gas gland, this transporter was expressed at a similar high level with 0.85 ± 0.27 × 10^6^ copies per 10 ng of total RNA. The second highest expression level was found for *slc2a10* with 90,486 ± 20,803 copies per 10 ng of total RNA and 38,228 ± 5885 copies per 10 ng of total RNA in uninfected yellow and silver eel gas gland, respectively. The only difference between uninfected yellow and silver eel gas gland tissue in the expression of members of the *slc2* family was detected for *slc2a2*. In yellow eels, 5020 ± 766 copies per 10 ng of total RNA were found for *slc2a2*, while in silver eel only 837 ± 94 copies per 10 ng of total RNA were detected (*P* < 0.05).

Of the *slc5* family of glucose transporters, *slc5a11* was expressed with 25,990 ± 5631 copies per 10 ng of total RNA in yellow eels, and in silver eel tissue a similar value was detected with 17,318 ± 407 copies per 10 ng of total RNA (Fig. 1b). Only *slc5a1* was expressed at a lower level in silver eels with only 2712 ± 406 copies per 10 ng of total RNA (Fig. 1b).

The sodium-dependent glucose transporter *kiaa1919* was also detected with 44,589 ± 12,556 copies per 10 ng of total RNA in uninfected yellow eel gas gland, and there was no difference in expression compared to uninfected silver eels (Fig. 1b).

Looking at the influence of the nematode infection on the distribution of the glucose transporters in gas gland tissue for the transporter expressed at the highest level (*slc2a1b*), no difference could be detected between uninfected and infected yellow or silver eel gas gland. In infected yellow eels, expression of *slc2a6* was significantly higher than in silver eels, but this glucose transporter was also expressed at a lower level, i.e., at a level of about 1000 copies per 10 ng of total RNA (Fig. 1a).

**Fig. 2:**
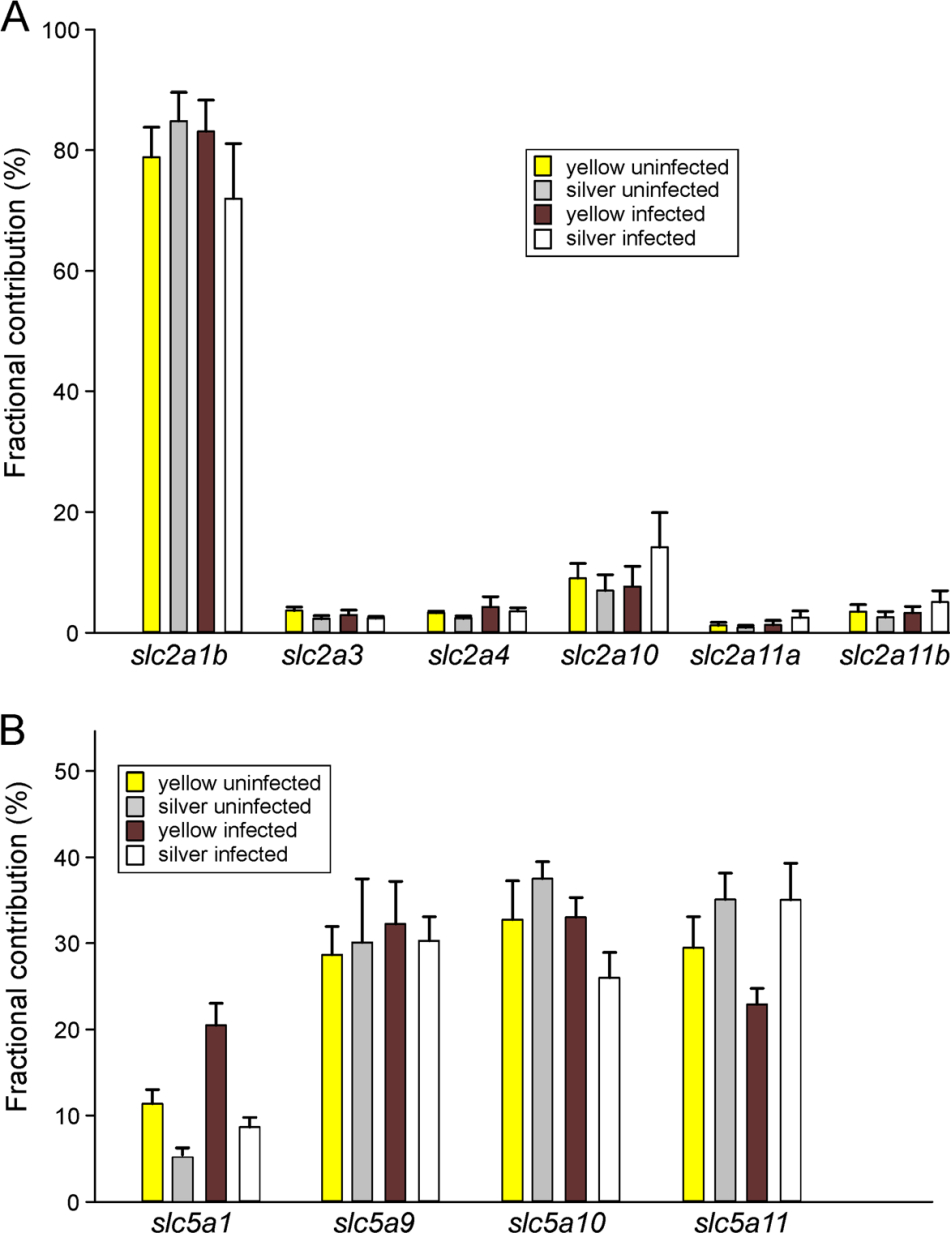
Fractional contribution (in %) of the different glucose transport transcripts of the *slc2* family (**a**) and of the *slc5* family (**b**) of glucose transporters in gas gland tissue of uninfected and infected yellow and silver eels. In Fig. 2a, *slc2* members contributing less than 1% to total SLC2 transcripts have been omitted for clarity. Mean ± S.E.M.; *N* = 5 for each group

Plotting the fractional contribution of the different glucose transport transcripts (Fig. 2) revealed that within the *slc2* family, *slc2a1b* with about 80% by far exceeded the expression level of all other family members, and there was no significant difference between yellow and silver eels and uninfected and infected eels (Fig. 2a). The second important transporter was *slc2a10* which contributed between 6.9 and 9.0% to total expression, and in infected silver eels even a value of 14.1% was found. The contribution of *slc2a2*, *5*, *6*, and *8* was even below 1% in gas glands of all experimental groups. No significant difference in the fractional contribution of the various glucose transporters was found between yellow and silver eels and uninfected and infected eels.

The fractional contribution of the different *slc5* family members was much more uniform (Fig. 2b), and in uninfected yellow eel gas gland tissue, each of *slc5a9*, *slc5a10*, and *slc5a11* contributed close to about 30% to the total transcript number, leaving 11.4 ± 1.6% for *slc5a1*. There was no significant difference in the expression level of *slc5* family members between yellow and silver eels and uninfected and infected eels.

**Fig. 3:**
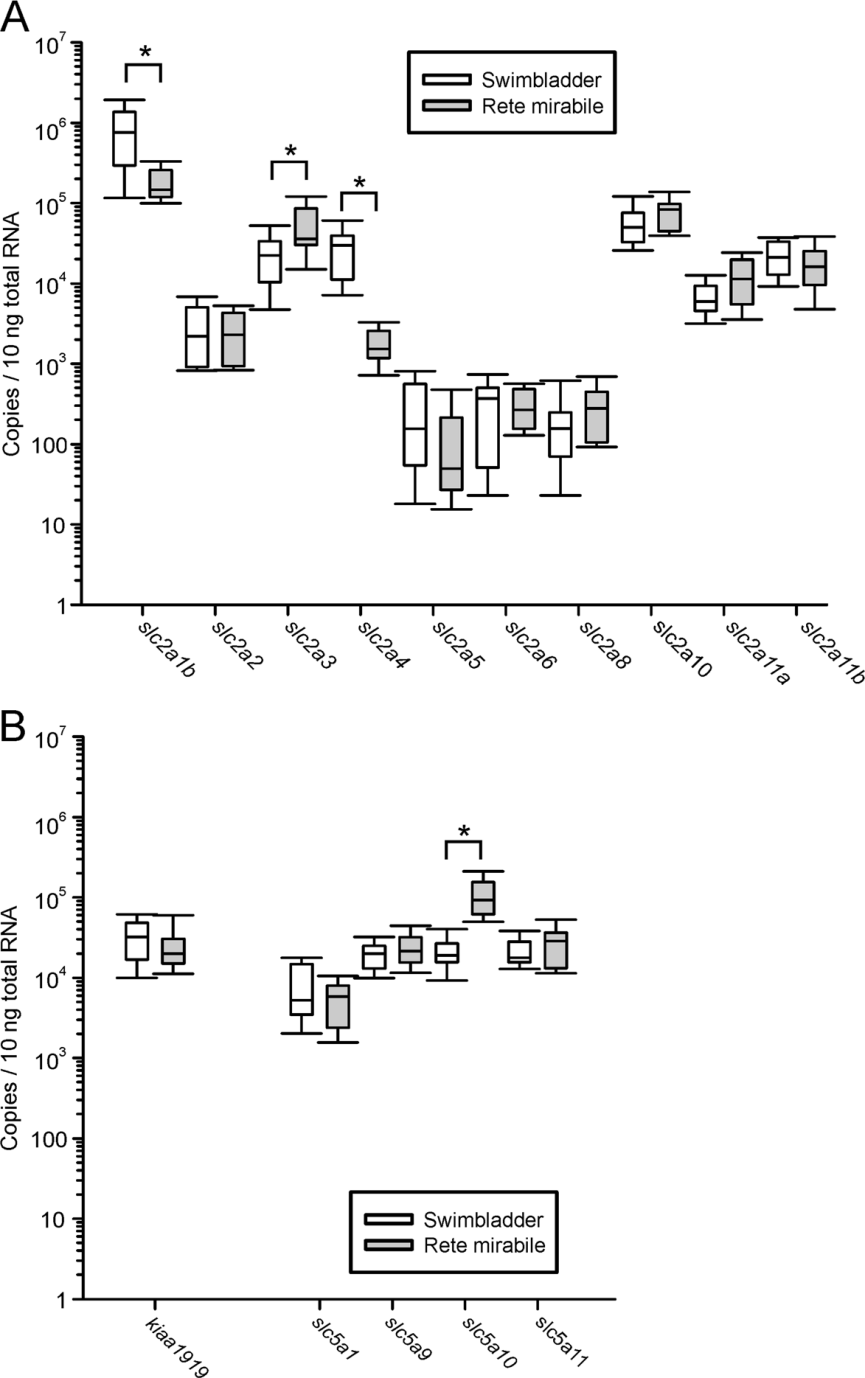
Number of mRNA copies of various members of the *slc2* (**a**) and the *slc5* (**b**) family of glucose transporters, and of *kiaa1919* (**b**) per 10 ng total RNA in rete mirabile tissue of European eels. For comparison, the respective expression of the transporters in gas gland tissue (overall mean of all samples) has been included. Due to the large differences in the copy number, data are presented on a log scale. *N* = 13 for the rete mirabile; *N* = 20 for gas gland samples. Bracket indicates significant differences between groups, *P* < 0.05

Testing the expression of glucose transporters in the rete mirabile of the swimbladder revealed that the same transcripts already detected in gas tissue were also expressed in rete tissue. Analyzed were three uninfected yellow eels, five uninfected silver eels, and five infected silver eels. No significant differences could be detected for any of the transcripts between these three groups. Therefore, all samples were combined (*N* = 13). Figure 3 presents the expression level of all glucose transporters detected in the rete in comparison to the overall mean values calculated for gas gland tissue. In the rete, *slc2a1b* was also the *slc2* family member expressed at the highest level, but this value, with 191,300 ± 25,640 copies per 10 ng of total RNA, was significantly lower than the expression in gas gland tissue (Fig. 3a). In addition to *slc2a10* which was expressed at the second highest level, *slc2a3* was expressed at almost the same level with 58,429 ± 12,784 copies per 10 ng of total RNA. *slc2a5*, *6*, and *8*, which were very low in gas gland tissue, also were expressed at the lowest level in the rete mirabile.

Of the *slc5* family, *slc5a1* was expressed at the lowest level with only 5646 ± 989 copies per 10 ng of total RNA, significantly lower than the other three members of this family. *slc5a10* was expressed at the highest level with 113,536 ± 18,222 copies per 10 ng of total RNA. For *slc5a9* and *slc5a11*, 25,136 ± 3654 and 28,208 ± 4286 copies were detected, respectively (Fig. 3b).

*Kiaa1919* was expressed at a level of 27,093 ± 5424 copies per 10 ng of total RNA (Fig. 3b), and this level was not different from expression values obtained for gas gland tissue.

**Fig. 4:**
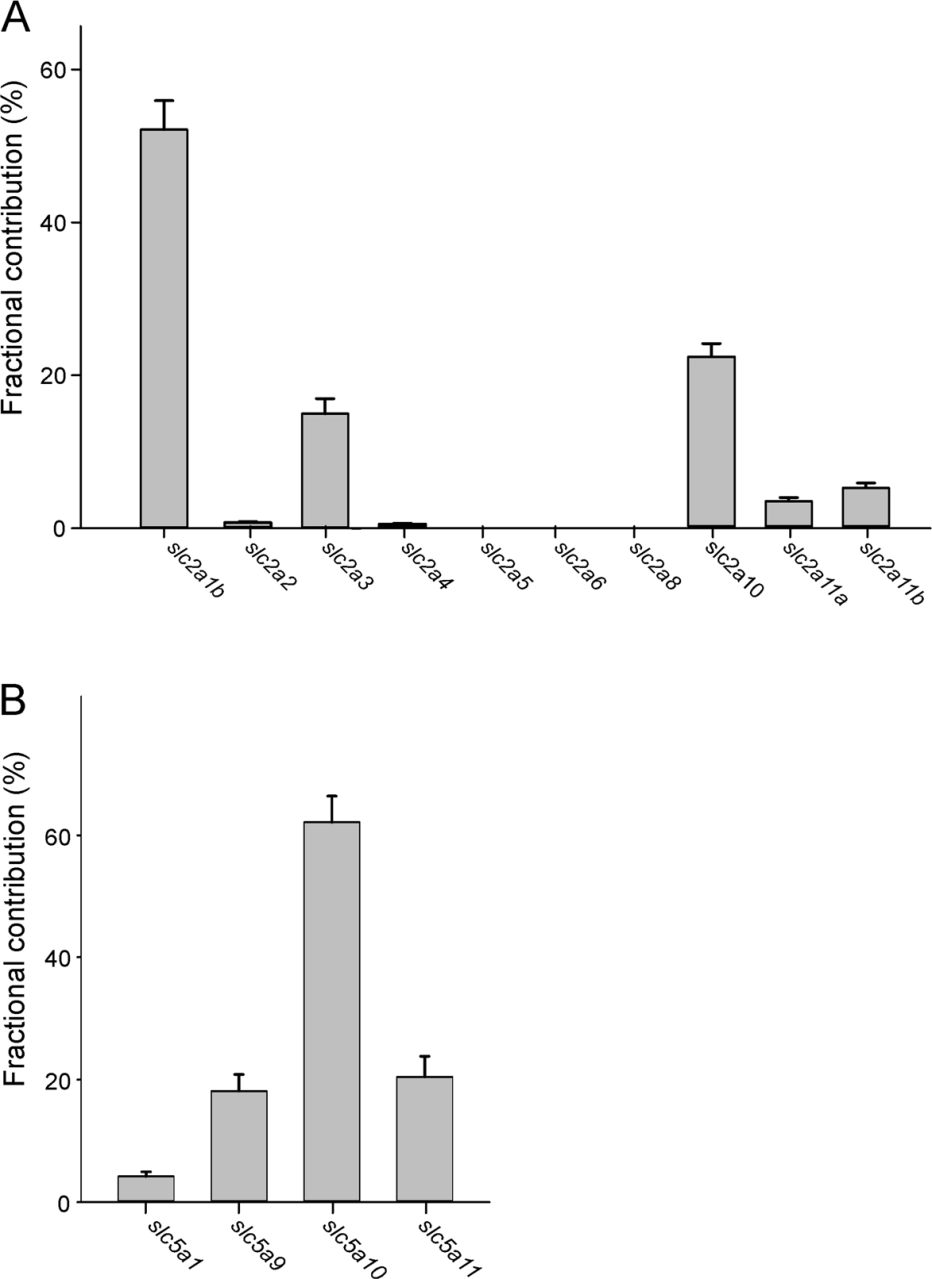
Fractional contribution (in %) of the different glucose transport transcripts of the *slc2* family (**a**) and of the *slc5* family (**b**) of glucose transporters in the rete mirabile of European eels. Mean ± S.E.M.; *N* = 13

The fractional contribution of the different glucose transport transcripts in the rete revealed significant differences to the picture obtained from gas gland tissue. Of the *slc2* family, *slc2a1b* was again the most important member, but it contributed to about 52 ± 4% to the total expression of this family, and *slc2a10* and *slc2a3* added another 22 and 15%, respectively (Fig. 4a), so that these three transcripts made up 89% of all transcripts of the *slc2* family. Of the *slc5* family, *slc5a10* was by far the most important member, making up about 62 ± 4% of the total expression (Fig. 4b).

**Fig. 5:**
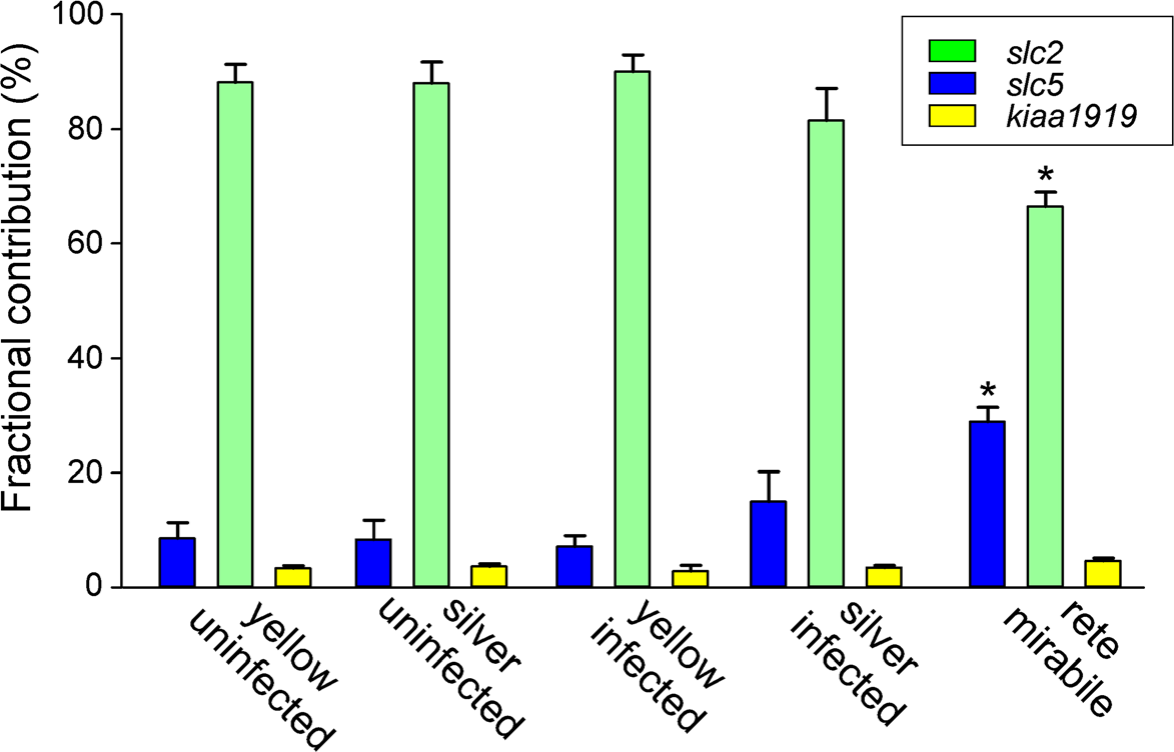
Comparison of the total expression level of members of the *slc2* family, the *slc5* family, and *kiaa1919* in gas gland tissue of uninfected and infected yellow and silver eel gas gland tissue and in the rete mirabile. Mean ± S.E.M.; gas gland tissue, *N* = 5 for each group; rete mirabile, *N* = 13. *indicates significant differences between gas gland tissue and rete mirabile, *P* < 0.05

Differences in the glucose transporter expression between gas gland tissue and rete mirabile were also obvious when comparing the total contribution of the two transporter families to all analyzed glucose transporter transcripts (Fig. 5). While in gas gland tissue the *slc2* family made up between 81 and 90% of the total glucose transporter transcripts, in the rete, they contributed only 66 ± 2.5%. The contribution of the *slc5* family in turn was significantly elevated compared to gas gland tissue. The contribution of glucose transporter *kiaa1919* varied between 2 and 5% in gas gland tissue and in the rete, and there was no difference between the different tissues and samples.

## Discussion

In fish, a tissue specific distribution of members of the SLC2 family of glucose transport proteins has been reported, and most studies focus on the first four members of this family, i.e., *slc2a1-4* (alias: *glut1-4*) (Hall et al. 2014). In fish, a genome duplication resulted in the presence of gene paralogs (Taylor et al. 2001; Meyer and Van de Peer, 2017), which may have been retained as in the case of HIF-*α* proteins in cyprinids (Rytkönen et al. 2013), but also may have been lost in evolution. Accordingly, we found duplicates of some of the glucose transporters, but not of all. Our genome search identified a number of additional fragments of some glucose transport proteins, but because they were incomplete and could not result in the production of a functioning transport protein, they were omitted. *Slc2a1b (glut1*) appears to be expressed ubiquitously (Hall et al. 2004; Hall et al. 2014). In eel gas gland tissue, *slc2a1b* (*glut1*) is by far the most dominant member of the *slc2* family, and this has also been reported for the Atlantic cod (Hall et al. 2004; Hall et al. 2014). Relative to total RNA, the number of *slc2a1b* (*glut1*) transcripts was 6-fold higher in eel gas gland tissue than in the rete mirabile. In fugu gas gland tissue, high expression of *glut1a* was detected by RT-PCR and in situ hybridization (Munakata et al. 2012). In Atlantic cod, expression of this transporter was also exceptionally high in gas gland tissue compared to other tissues (Hall et al. 2014), and the number of *glut1* transcripts coincided with the rate of glucose consumption (Clow et al. 2016), supporting the notion that gas gland tissue has an exceptionally high rate of glucose turn over. In the gas gland, glucose is required for lactate production in the glycolytic pathway and CO_2_ production in the pentose phosphate pathway. Both metabolites are required for acidification of the blood in order to switch on the Root effect, which in turn is essential for filling the swimbladder with oxygen (Pelster and Weber 1991; Pelster and Randall 1998; Pelster 2001).

SLC2a2 (GLUT2) has been found in liver of rainbow trout *Oncorhynchus mykiss* (Panserat et al. 2001), and in liver, but also in kidney and intestine of Atlantic cod *Gadus morhua* (Hall et al. 2006; Hall et al. 2014). In sea bass *Dicentrarchus labrax*, it has also been detected in larger amounts in brain tissue (Terova et al. 2009), but in eel swimbladder tissue, the *slc2a2* transcript contributed less than 1% to total *slc2* transcripts, suggesting that it does not play an important role in this tissue.

Similarly, *slc2a3* (*glut3*) and *slc2a4* (*glut4*) transcripts are expressed at a low level in gas gland cells. The SLC2a4 (GLUT4) transport protein is considered to be insulin regulated in fish (Capilla et al. 2002; Capilla et al. 2004; Diaz et al. 2009; Polakof et al. 2012; Marin-Juez et al. 2014). Our data therefore suggest that insulin is not involved in glucose uptake in gas gland tissue.

While many studies on SLC2 proteins in fish so far focused on SLC2a1-4, in swimbladder gas gland tissue *slc2a10* (*glut10*) contributed between 6.9 and 9.0% to total transcript level of *slc2* family members, and this was the second highest expression level of all analyzed members of this family. In fugu, *glut11c2* has been detected by RT-PCR, but not by in situ hybridization (Munakata et al. 2012). The functional significance of this transporter, however, remains unclear so far. *Slc2a10* and *11* have been found in the human genome, but even in mammals, the role of these transport proteins remains unclear (Mueckler and Thorens 2013).

Sodium-dependent glucose transporters (*slc5*, alias *sglt*) are most important in gut and kidney (Wright and Turk 2004), but a very low expression level was also reported for gas gland tissue of fugu (Munakata et al. 2012), and compared to the *slc2* transporters in the European eel, they contributed only about 10% to the total transcripts of glucose transporters. Three members of the *slc5* family (*slc5a9*, *10*, and *11*) appeared to be equally important, contributing about 30% each to total *slc5* transcripts, leaving about 10% for *slc5a1. slc5a1* (*sglt1*) has been identified in rainbow trout kidney (Sugiura et al. 2003; Conde-Sieira et al. 2013), hindbrain and hypothalamus (Otero-Rodino et al. 2015), and intestine (Polakof et al. 2010; Polakof and Soengas 2013). The swimbladder originates as an outgrowth of the esophagus and therefore is a derivative of the gut, so presence of sodium-dependent glucose transporters appears plausible.

Unfortunately, very little is known about the other three members of the *slc5* family (*slc5a9*-*11*), which are transcribed at a higher level in gas gland tissue. SLC5a9 (SGLT4) has been isolated from human small intestine, and it is also present in the kidney. In expression studies, it was characterized as a low affinity transporter, transporting sodium and various hexoses like mannose, glucose, and fructose (Tazawa et al. 2005). SLC5a10 (SGLT5) has been identified in human kidney and has also been shown to transport various hexoses in a sodium-dependent manner (Grempler et al. 2012; Fukuzawa et al. 2013). SLC5a11 (alias: SGLT6, SMIT2, KST1) has been shown to transport not only sodium and glucose but also sodium and *myo*-inositol. The mammalian SMIT2 protein has been reported to be sensitive to phlorizin, typical for SLC5 glucose transport proteins, and to transport *myo*-inositol even more effectively than glucose when expressed in *Xenopus* oocytes or in purified membrane preparations (Coady et al. 2002; Aouameur et al. 2007). The transport characteristics of the eel SLC5 proteins have not been analyzed as yet.

In addition to these well-known families of glucose transporters, the sodium-dependent glucose transporter *kiaa1919* (alias *NaGlt1*) was detected in gas gland tissue. KIAA1919 has been detected and characterized in rat kidney (Horiba et al. 2003; Nawata et al. 2015). Although glucose transport of this transporter is sodium dependent and sensitive to phlorizin, the low sequence identity with members of the SLC2 and the SLC5 family (< 22%) led the authors to conclude that this is a separate family of glucose transport proteins (Horiba et al. 2003). Compared to the *slc2* and the *slc5* family of glucose transporters, it was expressed at a low level, and there was no difference in the expression of *kiaa1919* between yellow and silver eel gas gland. Even in mammals very little is known about this transporter, and this appears to be the first report on this transporter in fish. The physiological significance of this transporter remains to be addressed.

By including silver eel tissue and also tissues from eels with swimbladders infected with the nematode *Anguillicola crassus*, we were also able to address the influence of silvering and of the nematode on the transcription pattern of *slc2* and *slc5* glucose transporters. In a previous transcriptomic study using unperfused swimbladder tissue, significant differences were detected for some *slc2* transporters (*slc2a3*, *5*, and *6*; (Pelster et al. 2016)). We confirmed significant differences in the transcript level of *slc2a2* and *slc2a6*, but our comparative analysis revealed that these *slc2* members are expressed at a very low level, i.e., they contributed less than 1% to total *slc2* transcription (i.e., *slc2a2*, *5*, *6*, and *8*). The physiological significance of these differences therefore remains questionable. In contrast to the European eel, in fugu a high level of *glut6* transcripts was detected by RT-PCR and by in situ hybridization (Munakata et al. 2012), suggesting that species specific differences in glucose transport mechanisms may exist. The most important *slc2a1b* transcript made up about 80% of all *slc2* transcripts in yellow and silver eel gas gland, irrespective of the infection status. Similar results were obtained for the second most important transcript, *slc2a10*, contributing about 7–9% to the *slc2* transcripts in all four groups. We therefore conclude from our data, that neither the silvering nor the infection of the swimbladder with the nematode *Anguillicola crassus* caused a significant modification of the transcription of *slc2* family members. In Atlantic cod, glucose turnover has been shown to be related to the mRNA expression of *slc2a1* (Clow et al. 2016). Our data suggest, however, that in the European eel, the adjustment of gas gland tissue glucose metabolism to the spawning migration and the severe changes in hydrostatic pressure associated with the daily vertical migrations in the open ocean (Aarestrup et al. 2009; Righton et al. 2016; Schabetsberger et al. 2016) do not require transcriptional modification of glucose transporters. This could imply that in European eel, the rate of glucose metabolism of gas gland cells is not correlated to the mRNA expression level of glucose transport proteins. Translational activity may play a more important role, or the activity of glucose transport proteins may be regulated. Vagal control of gas gland metabolism has repeatedly been confirmed, and vagotomy abolishes gas secretion (Pelster 1995b; Nilsson 2009; Smith and Croll 2011).

With respect to sodium-dependent transporters in gas gland tissue, high levels of *slc5a1* have been found in infected yellow eel, significantly higher than in uninfected silver eels. It is possible that the thickening of the gas gland tissue in infected swimbladders (Nimeth et al. 2000; Würtz and Taraschewski 2000) caused a modification of glucose transport, and an elevated transcription of glycolytic enzymes has indeed been observed in infected yellow eel gas gland tissue (Pelster et al. 2016). In addition, the infection significantly influenced the transcriptional changes observed during silvering (Pelster et al. 2016), possibly explaining the differences observed between infected silver and infected yellow eels.

An analysis of glucose metabolism of rete capillaries in vitro surprisingly revealed, that although the tissue faces high oxygen partial pressures, anaerobic metabolism dominates. Similar to gas gland cells, lactate is the main end product of glucose metabolism, accounting for more than 95% of the glucose uptake in rete tissue (Rasio 1973). Comparing *slc2*, *slc5*, and *kiaa1919* transcript levels of gas gland tissue with the levels measured in the rete mirabile; however, significant differences were detected. Of the members of the *slc2* family, *slc2a1b* contributed close to 50% to the overall transcript level, which was much less than its contribution in gas gland tissue. Together with *slc2a3* and *slc2a10*, these three transcripts made up close to 90% of the total *slc2* transcripts. Accordingly, while in gas gland tissue, *slc2a1b* was almost exclusively responsible for glucose transport, in rete tissue, these three transporters significantly participate in glucose uptake. As already observed in gas gland tissue, *slc2a2*, *5*, *6*, and *8* were almost negligible in their transcript level.

In rete tissue, the transcriptional contribution of the various *slc2* members was more equally distributed than in swimbladder tissue, and the sodium-dependent glucose transporters were much more important. While in uninfected yellow and silver eel gas gland tissue, the sodium-dependent transporters contributed less than 10% to total glucose transporters, in the rete mirabile, their contribution increased to 28%, suggesting that in the rete, sodium-dependent glucose transport was much more important than in gas gland tissue. As in gas gland tissue, *slc5a1* was expressed at a low level. In contrast to the results obtained from gas gland tissue with respect to *slc5a9*-*11*, in the rete, *slc5a10* was by far the dominating sodium-dependent glucose transporter, contributing 62 ± 4% to the total *slc5* transcripts measured. In mammals, SLC5a10 (SGLT5) expression has only been shown in the kidney, and it appears to transport various monosaccharides (Grempler et al. 2012; Fukuzawa et al. 2013). In swimbladder tissue, only glucose transport appears to be relevant, so far no other hexose has been implicated in swimbladder metabolism. Our data suggest that in the rete, glucose transport is much more dependent on sodium co-transport than in gas gland tissue. Considering the high rate of glucose consumption in gas gland cells, it could be possible that a large fraction of the glucose is in fact taken up by these cells, leaving only low blood glucose concentrations in venous blood returning to the rete mirabile. Thus, sodium-dependent glucose uptake could be required to assure proper supply of nutrients to rete mirabile endothelial cells.

In all gas gland samples, irrespective of the infection status, and in the rete mirabile, *kiaa1919* transcripts contributed less than 5% to total glucose transporter transcripts, and there was no difference between the different samples. Accordingly, the contribution of this transporter to glucose uptake did not appear to be of great importance, and the physiological meaning of this transporter remains to be analyzed.

In conclusion, the results of our study show that in swimbladder gas gland cells of the European eel, *slc2a1b* (*glut1*) was by far the most dominant member of all expressed glucose transporters. However, expression of the members of glucose transporter families *slc2* and *slc5* was hardly affected by silvering, which has been shown to enhance swimbladder function and thus must be connected to an elevated metabolic activity and acid production. In addition, the infection of the swimbladder with the nematode *Anguillicola crassus* had no severe effect. This was surprising because an infection of the swimbladder with the nematode significantly impairs acid and gas secretion (Würtz et al. 1996) and thus metabolism of gas gland cells. Taken together these data suggest that, in contrast to cod (Hall et al. 2014), in European eel, glucose metabolism in gas gland cells is not strictly correlated to the mRNA expression level of glucose transport proteins. Obvious differences were established in the expression of glucose transport family members between the rete mirabile and the gas gland. Rete tissue is much more dependent on sodium-dependent glucose uptake, which may be related to the extremely high rate of glucose uptake by gas gland cells, leaving only low levels of glucose in the venous return to the rete.
